# Does Twitter Trigger Bursts in Signature Collections?

**DOI:** 10.1371/journal.pone.0058252

**Published:** 2013-03-06

**Authors:** Rui Yamaguchi, Seiya Imoto, Masahiro Kami, Kenji Watanabe, Satoru Miyano, Koichiro Yuji

**Affiliations:** 1 Human Genome Center, The Institute of Medical Science, The University of Tokyo, Tokyo, Japan; 2 Division of Social Communication System for Advanced Clinical Research, The Institute of Medical Science, The University of Tokyo, Tokyo, Japan; 3 Center for Kampo Medicine, Keio University School of Medicine, Tokyo, Japan; 4 Department of Internal Medicine, Research Hospital, The Institute of Medical Science, The University of Tokyo, Tokyo, Japan; Tokai University, Japan

## Abstract

**Introduction:**

The quantification of social media impacts on societal and political events is a difficult undertaking. The Japanese Society of Oriental Medicine started a signature-collecting campaign to oppose a medical policy of the Government Revitalization Unit to exclude a traditional Japanese medicine, “Kampo,” from the public insurance system. The signature count showed a series of aberrant bursts from November 26 to 29, 2009. In the same interval, the number of messages on Twitter including the keywords “Signature” and “Kampo,” increased abruptly. Moreover, the number of messages on an Internet forum that discussed the policy and called for signatures showed a train of spikes.

**Methods and Findings:**

In order to estimate the contributions of social media, we developed a statistical model with state-space modeling framework that distinguishes the contributions of multiple social media in time-series of collected public opinions. We applied the model to the time-series of signature counts of the campaign and quantified contributions of two social media, i.e., Twitter and an Internet forum, by the estimation. We found that a considerable portion (78%) of the signatures was affected from either of the social media throughout the campaign and the Twitter effect (26%) was smaller than the Forum effect (52%) in total, although Twitter probably triggered the initial two bursts of signatures. Comparisons of the estimated profiles of the both effects suggested distinctions between the social media in terms of sustainable impact of messages or tweets. Twitter shows messages on various topics on a time-line; newer messages push out older ones. Twitter may diminish the impact of messages that are tweeted intermittently.

**Conclusions:**

The quantification of social media impacts is beneficial to better understand people’s tendency and may promote developing strategies to engage public opinions effectively. Our proposed method is a promising tool to explore information hidden in social phenomena.

## Introduction

Much commentary on the impact of social media, such as Twitter, on societal, political, and medical events exists [1]–[10]. To measure the causal effect of social influence online, experimental studies have been attempted [11]. But previous researches suggested that online communication may not to be an effective medium for social influence [12] and the quantification of such influences is a difficult undertaking [13], [14]. One reason is that not all active participants in social media discussions take action and those who passively read text may act. Another is that because of the multiple social media, including Internet forums and blogs, it is hard to distinguish the contributions of each.

To measure such effects on a time-series of collected public opinions, we developed a statistical model that estimates the contributions of multiple social media. We applied it to the data of a recent signature-collecting campaign to oppose a medical policy in Japan and succeeded in detecting the impacts of Twitter and an Internet forum.

On November 20, 2009, the Japanese Society of Oriental Medicine and some patients started a website [15] to gather signatures from the public to oppose a medical policy of the Government Revitalization Unit [16] to exclude a traditional Japanese medicine, “Kampo,” from the public insurance system. The signature count showed a series of aberrant bursts from November 26 to 29, 2009. In the same interval, the number of messages on Twitter including the keywords “Signature” and “Kampo,” increased abruptly. Moreover, the number of messages on an Internet forum that discussed the policy and called for signatures showed a train of spikes. These observations motivated us to estimate the impacts of the two social media.

## Methods

### Data Sets

**Figure 1:**
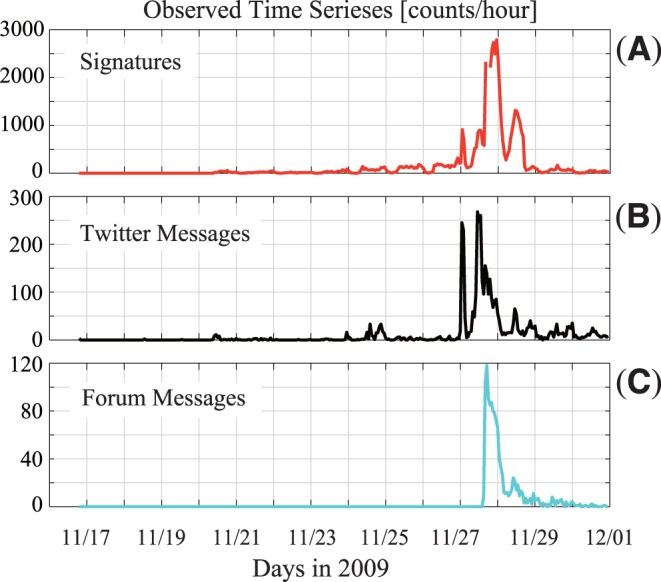
The observed numbers of signatures and messages of social media. The observed hourly-counted numbers of (A) signatures (*y_n_*), (B) Twitter messages including “Kampo” and “Signatures” (*ϕ_n_*), (C) Internet-forum messages (*ω_n_*).

Three observed time-series data are used in this analysis: hourly counts of signatures, *y_n_* (Figure 1A); Twitter messages, *ϕ_n_* (Figure 1B); and messages on the Internet forum, *ω_n_* (Figure 1C). The time index *n* (*n* = 1,…,*N*) indicates the *n*th hour, starting from 19∶00 on November 16, 2009 (*n* = 1) and ending at 23∶00 on November 30, 2009 (*n* = *N* = 341). The original messages on Twitter were obtained from the web site [17] by querying messages including the both keywords “Signature” and “Kampo,” (in Japanese). The original messages on the Internet forum were publicly available and obtained from the web site [18]. We note that *y_263_* and *y_264_*, which correspond to the two hours from 17∶00 to 19∶00 on November 27, are set as missing observations for the analysis in order to avoid a harmful influence for the estimation because a malfunction of the web server for the signature collection campaign that was probably induced by a surge of accesses to the site largely impeded to collect the signatures; the actual numbers of signatures were only 32 and 588 for the two time points, which were much smaller than those of just before and after the period. Our analysis method can deal with missing observations properly by a Bayesian estimation with Kalman filter algorithm.

### Decomposition Model for Signatures

The goal of this study is to estimate the amount of contributions to the signature collecting campaign from those who were affected by either the Twitter or the Internet forum messages, and to discuss the modes of impacts of each social media based on the estimated contribution profiles. However, it is a challenge because we cannot directly observe the behaviors of contributors behind the Internet, and thus it is obviously hard to distinguish information sources to motivate each of them. In order to tackle such a difficulty, we employ a power of mathematical modeling; we develop a stochastic time-series model that equips components explaining the contributions to the observed time-series of the number of signatures from those who were affected by Twitter, the Internet forum, and other unknown information sources (Equation 1). By applying the model to the observed time-series data, we can estimate the unobserved contribution profiles.

Before explaining details of the model equations, we briefly describe our premise for the model building as follows: 1. The information sources, i.e., Twitter, the Internet forum, and some other unknown sources, affected to the contributors, and those effects were mutually exclusive, that is, each of contributors was affected from only one of the information sources. This assumption makes the model simple and easy for interpretations, though it neglects possible interactions among the media; we discuss the limitation of this modeling later. 2. Each of the effects of Twitter and the Internet forum can be represented by a product of an activity and an effectiveness of the media. We assume that the number of the observed messages per hour in each of the media represents the activity and that the effectiveness is time-varying. 3. The effect from the unknown sources has a smooth profile.

To estimate the impact of the Twitter and Internet forum messages on the number of signatures, we assume that the time-series can be modeled by.
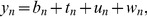
(1)where *b_n_, t_n_, u_n_,* and *w_n_* are the baseline effect, Twitter effect, Forum effect, and observation noise (residual component), respectively. The observation noise component is modeled by a normal distribution: 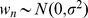. The other components are explained below.

### Baseline Effect

The baseline effect *b_n_* is a component representing a smooth variation in the number of signatures collected from people influenced by effects other than Twitter and the Internet forum. The component is modeled by the second-order stochastic difference equation [19]:
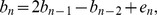
where 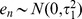; the smoothness of variations in the time-series of *b_n_* is modeled by the similarity of slopes in a sequence of time-points, i.e., 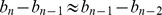.

### Twitter Effect

The Twitter effect *t_n_* is a component of the contributions of people affected by the Twitter signature collection campaign. It is assumed to be proportional to the number of messages *ϕ_n_* as follows:
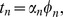
where 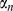 is a time-varying coefficient modeled by the first-order stochastic difference equation,



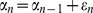
with 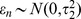; 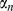 represents an effectiveness of the messages at time *n*.

### Forum Effect

The forum effect *u_n_* is a component of the contributions of people affected by the Internet forum in signature collection campaign; it is modeled in the same manner as the Twitter effect, with the number of the messages 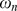 in the Internet forum, as follows:
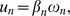
where



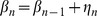
with 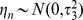; 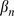 represents an effectiveness of the messages at time *n*.

### Estimation

To estimate each of the components *b_n_, t_n_, u_n_,* and *w_n_* in the decomposition model of the observed time-series of signatures (*y_n_*) (Equation 1), we convert the above equations into a state-space model form [19] and then decompose the time-series by estimating the conditional expectation values of state vectors 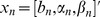 with the Kalman filter and the fixed interval smoother algorithms. In the following analysis, we discuss the decomposed components based on smoothing estimates of the state vectors, i.e., conditional expectation values given the entire time-series observation data. The parameters 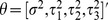 are estimated by maximizing the marginal likelihood.

## Results

The signature count exhibited small variations until November 25; however, from November 26 to 29, it showed a series of aberrant bursts (Figure 1A). In the same interval, the number of messages on Twitter [17], including the keywords “Signature” and “Kampo,” (in Japanese) increased abruptly (Figure 1B). Moreover, the number of messages on an Internet forum [18] that discussed the policy and called for signatures showed a train of spikes (Figure 1C).

**Figure 2:**
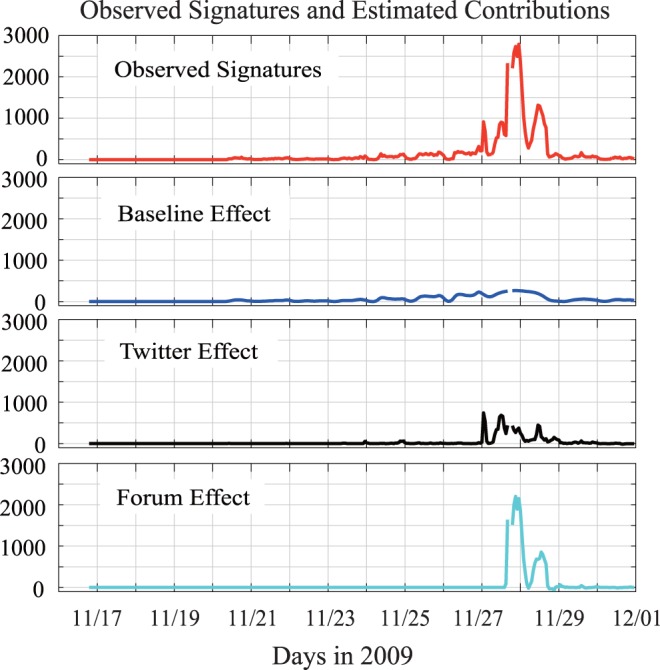
The observed signatures and decomposed profiles of each effect. The observed hourly-counted numbers of signatures (*y_n_*) and the estimated hourly-counted Baseline effect (*b_n_*), Twitter effect (*t_n_*), and Forum effect (*u_n_*) by the decomposition model are shown. The estimated profiles are based on the smoothing estimates from Kalman smoother.

We quantified the impacts of social media on the campaign using the statistical model. A total of 95,362 signatures were gathered on the web. 43,190 were obtained in only four days–from Nov. 27 to Nov. 30, 2009. We decomposed the time-series of signatures into a Twitter effect, Forum effect, and baseline effect­ (Figure 2); the latter is the contributions of people affected by other implicit sources. We assume that the number of message­s at each time point (Figures 1B, 1C) measure the activities of the two media and that these activities influence the decisions of participants; the effectiveness of these activities are expressed as time-varying weights. In comparison to other models that include sub-set components of the full-set model (Equation 1), the full-set model had the best predictive power, which was evaluated by Akaike information criterion (data not shown).

**Figure 3:**
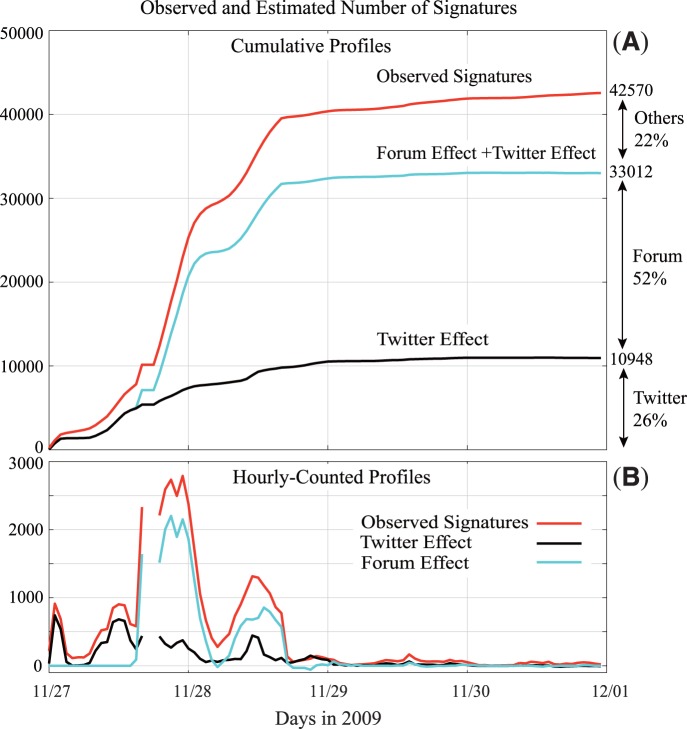
The observed signatures and estimated contributions from the two social media. (A) the cumulative and (B) the hourly-counted profiles of the observed signatures and the estimated Twitter and Forum effects.

The cumulative profiles of the observed number of signatures and estimated contributions of the Twitter and Forum effects suggest that the latter could explain a large portion of the observed signatures (78%) during the period (Figure 3A). The total contribution of the Twitter effect (26%) was smaller than that of the Forum effect (52%). These profiles also indicate that Twitter probably triggered the initial two bursts of signatures on November 27 and the Internet forum, most of the latter bursts (Figure 3B).

## Discussion

The first surge in signature numbers occurred on Nov. 27 between 1 and 3 AM, off-peak internet hours (Figure 1A). At the same time, Twitter user trends showed a sudden increase in the number of tweets including the words “Kampo shomei” (Kampo signature). These words were seldom tweeted before the petition, and the mass media had not yet picked up the story, suggesting that Twitter played a significant role in increasing the number of signatures of the first surge. Previous research suggested that Twitter has the power to disseminate information through networks of followers and a culture of “retweeting” [20] and this study confirmed that Twitter’s real-time, viral mode of communication effectively mobilized and amplificated a protest against the budget-slashing policies of the Japanese government. Of further interest to us is that rapid spread of messages occurred among anonymous Twitter users. Even among social networks, close relationships have a stronger behavioral effect on each other than do strangers [13], [21], [22]. Social mobilization in online networks might be significantly more effective than informational mobilization alone [10]. While Twitter has the potential to increase public awareness of various issues and to change social behaviors, the possibility of disseminating false information remains a key concern [23]. We must keep this in mind when utilizing Twitter to share health information among physicians, patients, and the public [21], [22].

Although the numbers of messages on both Twitter and the Internet forum were comparably small during the last burst of November 28, the estimated Forum effect was larger (Figure 2). Twitter usually shows messages on various topics on a time-line; newer messages push out older ones. Therefore, it is relatively hard to follow long-term trends and recuperate messages that disappear. As a result, Twitter ­may diminish the impact of messages that are tweeted intermittently. On the other hand, an Internet forum usually discusses a particular topic and new readers can follow past discussions easily; consequently, a few messages may be able to sustain a larger effect for a longer time than Twitter.

There are several limitations to our study. First, we analysed data tweeted in Japanese and limted to Japan only. The performance of our model may be biased and suffer. Second, the demographic of Twitter population that would tweet about “Kampo” may not represent the general population, especially the population that would provide their names and addresses for the petition. Third, we did not analyse tweets and signatures across geography. Creating a “mashup” [24], [25], which combines tweets’ location data with signatures’ addresses, would help improve the accuracy of the relationship between twitter and the number of signatures. Fourth, we ignored possible interactions between the media. There are difficulties to estimate such interaction effects because we could not obtain data that contain sufficient information for trajectories of the users. For example, we could not know twitter accounts of forum users who posted messages to the forum since the forum allowed users to post anonymously and almost all users were anonymous. If we can use such information, it may be useful to estimate some interaction effects in case there were actual interactions. Such information is hard to gather unless it was prospectively collected. It remains in our future works. Fifth, we assumed that each of tweets had the same impact; it was because we could not utilize sufficient information to differentiate the impacts of tweets, e.g., the numbers of followers of twitter users. This assumption probably prevented our estimation from accounting for the overall influence of tweets precisely because tweets from different individuals may have dissimilar reachability due to wide distributions of in-degree (‘followers’) and out-degree (‘friends’) of users and thus have varying impacts [26], [27]. Therefore we should consider the impacts of individual tweets to improve the accuracy of the estimation in our future work. For such a study, gathering the connectivity information among a huge number of users in a prospective manner would be required [27].

In conclusion, quantification of impacts of social media on a medical campaign is beneficial to better understand people’s tendency and may promote developing strategies to engage public opinions effectively. Our proposed method is a promising tool to explore big-data information [3] hidden in social phenomena.
